# Bioinspired 3D braided artificial ligament with human-like mechanical properties and self-healing capability

**DOI:** 10.3389/fbioe.2025.1701754

**Published:** 2025-10-23

**Authors:** Junnan Teng, Bingqian Li, Xiyang Zhao, Kunyang Wang, Lei Ren, Hong Xie, Xinbo Wang, Yilin Su, Luquan Ren

**Affiliations:** ^1^ Key Laboratory of Bionic Engineering (Ministry of Education), Jilin University, Changchun, China; ^2^ Institute of Structured and Architected Materials, Liaoning Academy of Materials, Shenyang, China

**Keywords:** bioinspired, artificial ligament, self-healing, shape memory alloy, braiding

## Abstract

**Introduction:**

Joint injuries, a major type of human musculoskeletal disorder, are often accompanied by soft tissue damage, and restoring ligament integrity is a key strategy for reconstructing joint function. However, existing artificial ligaments face a critical challenge: reconciling robust biomechanical performance with intrinsic self-healing capability, especially under cyclic loading and accidental overload conditions. Conventional materials like polyethylene terephthalate (PET) and polytetrafluoroethylene (PTFE) struggle with long-term durability, while emerging self-healing designs are limited by poor mechanical robustness and slow healing kinetics.

**Methods:**

This study developed a self-healing artificial ligament via 3D braiding of shape memory alloy (SMA, Ni_50.71_Ti_49.29_) wires and polyethylene (PE) fishing lines, mimicking the hierarchical structure of natural ligaments. The ligament was fabricated with a 1-over-1-under interlock configuration (6 carriers, 180° braid angle) and pre-tensioned (5% strain at 60 °C for 12 h) for structural stabilization. Differential Scanning Calorimetry (DSC), Dynamic Mechanical Analysis (DMA), and mechanical tests (hysteresis, stress relaxation, cyclic loading) were conducted to characterize its thermal and mechanical properties. Electrothermal recovery tests (3–5.5 W power input) evaluated self-healing performance, and a 3D-printed artificial hip joint was used to validate in-situ functionality.

**Results:**

DSC showed the SMA had a thermal hysteresis window of 24.8 °C (Ms=46.5 °C, Mf=27.2 °C, As=58.3 °C, Af=71.3 °C), and DMA revealed an “S”-type storage modulus curve during heating . After 1,000 s of cyclic loading, the self-healing ligament retained ∼73% of initial stress (vs. 37% for conventional ligaments) and had a lower energy dissipation ratio due to SMA’s low damping. Electrothermal tests showed maximum contraction rate increased with pre-strain, and 3–5.5 W power input enabled proportional contraction strain. In artificial hip tests, SMA activation restored ∼95% of initial joint laxity, reducing excessive rotational/translational motion by 26% and 12% respectively.

**Discussion:**

The hybrid SMA-PE design resolves the trade-off between biomechanical performance and self-healing: PE provides foundational tensile strength, while SMA enables electrothermal self-healing via phase transformation. The 3D braided structure replicates natural ligaments’ J-shaped stress-strain behavior, ensuring adaptability to dynamic joint movements. Compared to piezoelectric nanomaterial (PENM)-based designs (focused on proprioception), this ligament prioritizes mechanical stability and rapid self-healing, making it suitable for clinical rehabilitation and assistive devices. Future work will address limitations like wired power supply (via wireless modules) and long-term stability (via anti-degradation coatings).

## Highlights

• A biomimetic self-healing ligament design achieved by integrating SMA wires with fishing lines.
• The combination of SMA wires and polyethylene (PE) fibers endows the ligament with human-like mechanical properties and self-healing capability.
• Hierarchical 3D braiding confers low energy dissipation ratio and high stress retention performance to the artificial ligament.
• The self-healing ligament enhances joint stability and restores joint laxity even after severe damage.

## 1 Introduction

Joint injuries—particularly those involving ligament fatigue or damage—pose a major challenge in musculoskeletal medicine, as they severely compromise joint stability, mobility, and overall quality of life (Katz et al., 2021; Kaeding et al., 2015; Bhattacharjee et al., 2023). Such injuries arise from diverse causes, including acute trauma, repetitive mechanical stress, excessive workload, and degenerative conditions. If left untreated or inadequately repaired, they often progress to chronic pain, impaired motor function, and increased risk of osteoarthritis, imposing a substantial burden on global healthcare systems (De Bock et al., 2022; Ward et al., 2018; Wen and Lohmander, 2014). Restoring ligament integrity is therefore critical not only for functional recovery but also for preventing long-term complications, making it a focal area of research in rehabilitation engineering (Kw et al., 2019; Wang et al., 2015; Park et al., 2014; Su et al., 2024). Beyond their therapeutic utility, artificial ligaments also hold potential as advanced assistive devices and educational tools, enabling the simulation of physiological biomechanical properties for medical education and surgical training (Riener et al., 2004; McCloy and Stone, 2001).

Since the 1960s, significant efforts have been devoted to the development of commercial artificial ligaments using biocompatible materials (e.g., carbon fiber, Dracon) (Jenkins et al., 1977; Jackson and Arnoczky, 1993). While these materials exhibit good biocompatibility with human tissues and reduce the risk of adverse reactions, their biomechanical performance remains inferior to that of native ligaments. In recent years, high-strength polymers—such as those used in Gore-Tex™, 3 M Kennedy LAD™, and LARS™—have been adopted for ligament tissue regeneration and augmentation, providing enhanced joint stabilization (Dellestable et al., 2024; Winnisch et al., 2018; Migliorini et al., 2022). Despite decades of innovation in ligament repair, however, existing artificial ligaments suffer from notable limitations. Conventional materials like polyethylene terephthalate (PET) and polytetrafluoroethylene (PTFE) can meet the tensile strength and initial stability requirements for joint reconstruction but fail to ensure long-term durability (Zhang et al., 2025a; Wang et al., 2023; No et al., 2020). Under repeated loading cycles, these materials undergo creep, leading to gradual loosening and the inability to maintain required tension over time (Kaeding et al., 2015; Crawford et al., 2013). Additionally, current fabrication methods offer limited control over the spatial distribution of fibers, as well as the mechanical and structural properties of the final ligament. Furthermore, most existing artificial ligaments are designed exclusively for *in vivo* joint injury treatment and are not suitable for use in assistive artificial joints (Rinoldi et al., 2021; Puetzer et al., 2021).

Emerging research on self-healing artificial ligaments provides a promising solution to these challenges (Liu et al., 2021; Jao et al., 2016). By leveraging smart materials, self-healing ligaments are engineered to mimic the natural repair mechanisms of biological tissues. Current strategies—including thermally, chemically, and electrostatically driven self-healing systems—have shown potential in experimental settings. These materials, often modified with dynamic chemical bonds or supramolecular structures, can autonomously recover after damage, thereby extending ligament longevity and preserving functionality (Bin Ying et al., 2021; Li et al., 2016; Markvicka et al., 2018). Nevertheless, most existing self-healing approaches are constrained by limited mechanical robustness, slow healing kinetics, and poor scalability for clinical and educational applications (Zhang et al., 2025b; Pei et al., 2024).

Native ligaments possess unparalleled structural sophistication, derived from their hierarchical protein architecture. At the nanoscale, aligned collagen fibrils form the primary load-bearing units; these fibrils are further organized into progressively larger bundles at the microscale and macroscale (Puetzer et al., 2021; Stender et al., 2018; Provenzano and Vanderby, 2006). This hierarchical arrangement endows native ligaments with exceptional tensile strength and resilience. Moreover, the J-shaped stress-strain behavior of native ligaments enables energy dissipation at low strains and robust load transmission at high strains—critical for adapting to dynamic joint movements (Hearle et al., 1969; Nie et al., 2017). Another remarkable feature of native ligaments is their intrinsic self-healing capacity: upon microdamage, tenocytes are recruited to synthesize collagen precursors, and collagen fibrils are realigned along stress trajectories, ensuring functional recovery (Leong et al., 2020; Frank et al., 1999). Replicating these structural and functional features in synthetic systems remains a compelling yet formidable engineering challenge.

Building on the authors’ previous work—where 3D braiding was used to replicate the mechanical behavior of native ligaments—this study further develops a composite system of SMA wires and polyethylene (PE) fishing lines to impart self-healing capability to artificial ligaments, addressing the long-term durability limitations of traditional materials (Lu et al., 2022; Lu et al., 2024). Specifically, we propose a braided, bioinspired round artificial ligament that integrates SMA fibers with high-strength fishing lines. This design not only mimics the hierarchical structure and J-shaped mechanical response of native ligaments but also achieves self-healing via the shape memory effect of SMA fibers. When the ligament undergoes stress-induced elongation or relaxation, the SMA component can be activated via electrical stimulation to restore the ligament to its original state. To validate this design, we developed an artificial hip joint incorporating the self-healing ligament. Results show that SMA activation enhances joint stability, with post-recovery kinematics restoring 95% of the initial joint laxity. This system ensures rapid self-healing while maintaining mechanical integrity across multiple deformation cycles, making it suitable for both clinical applications and rehabilitation devices in joint injury treatment. Furthermore, the fabrication process is scalable and cost-effective, which can significantly enhance the overall durability and lifespan of artificial ligaments.

## 2 Materials and methods

### 2.1 Fiber selections

Polyethylene (PE) fishing lines with a diameter of 0.5 mm were employed in the braiding process. These lines feature a 9-strand multifilament structure, providing a cost-effective option with exceptional abrasion resistance and outstanding strength. The shape memory alloy fiber is commercially available Ni_50.71_Ti_49.29_ alloy with a diameter of 0.2 mm. The transformation temperatures are as follows: M_f_ is 27 °C, M_s_ is 46.5 °C, A_s_ is 58.3 °C, and A_f_ is 71.3 °C. The maximum electrically induced contraction strain is 5%.

### 2.2 Morphology of 3D braided structure

We utilized a braiding disc to fabricate the three-dimensional braiding structure. The strands refer to the number of threads used in the braiding process, and the braiding angle represents the angle between the threads of the same color during the braiding process. The specific braiding process is presented as follows.

Based on strands number and braiding angle requirements, lines of the same color are designated as exchange groups. During braiding, for each exchange group of the same color, the strand closest to the counterclockwise direction is swapped with the strand next to it in the clockwise direction. Then, the strand closest to the clockwise direction is swapped with the strand that was originally closest to the counterclockwise direction, completing one round of wire paths for an exchange group. Next, the first round of braiding is performed according to the color groups arranged by strand count, continuing until all exchange groups in the first round are completed. Afterward, the braiding process is restarted by cycling the color exchange groups initially positioned in the counterclockwise direction. Finally, the sample is successfully manufactured with the detailed designs and dimensions shown in Table 1.

**TABLE 1:** The detailed designs and dimensions of the self-healing ligament.

Parameter	Value	
Strands number	Fish line	4
	SMA	2
Braiding angles (°)	180 (A 180° braiding angle specifically means that the braided threads are arranged parallel to the ligament’s axial direction, consistent with the 1-over-1-under interlock configuration)	
Working length (mm)	200	
Diameter (mm)	Sample 1	1.42
	Sample 2	1.53
	Sample 3	1.43
	*Average*	1.46

### 2.3 Differential scanning calorimetry tests

The phase transition behavior and temperatures were characterized using a differential scanning calorimeter (TA DSC250, United States). The tests were conducted at a heating and cooling rate of 5 °C/min within a temperature range of 0 °C–120 °C.

### 2.4 Dynamic mechanical analysis

The dynamic mechanical properties were characterized using a dynamic mechanical analyzer (TA DMA850, United States). The tests were performed at a heating rate of 3 °C/min within a temperature range of 20 °C–120 °C.

### 2.5 Mechanical testing

The mechanical tests were performed through axial loading with a universal testing machine (Instron, United States). The samples were securely affixed within the machine grippers. The two ends of the artificial ligament are tightly secured to the fixtures to prevent relative slippage between the artificial ligament and the fixtures during the stretching process.

Hysteresis test: The ligaments are conducted by setting the loading displacement to certain displacement (5, 10, 15, 20, 25 mm) and then unloading to the initial position at different loading rates (10, 30, 50, 70, 90 mm/min).

Stress relaxation tests: The specimen was subjected to tensile strain at a rate of 50 mm/min until reaching a strain of 30%. Subsequently, the strain was held constant, and the variation in the rebound force of the specimen was recorded within a period of 1000 s.

## 3 Results

### 3.1 Composition of self-healing artificial ligament

**FIGURE 1:**
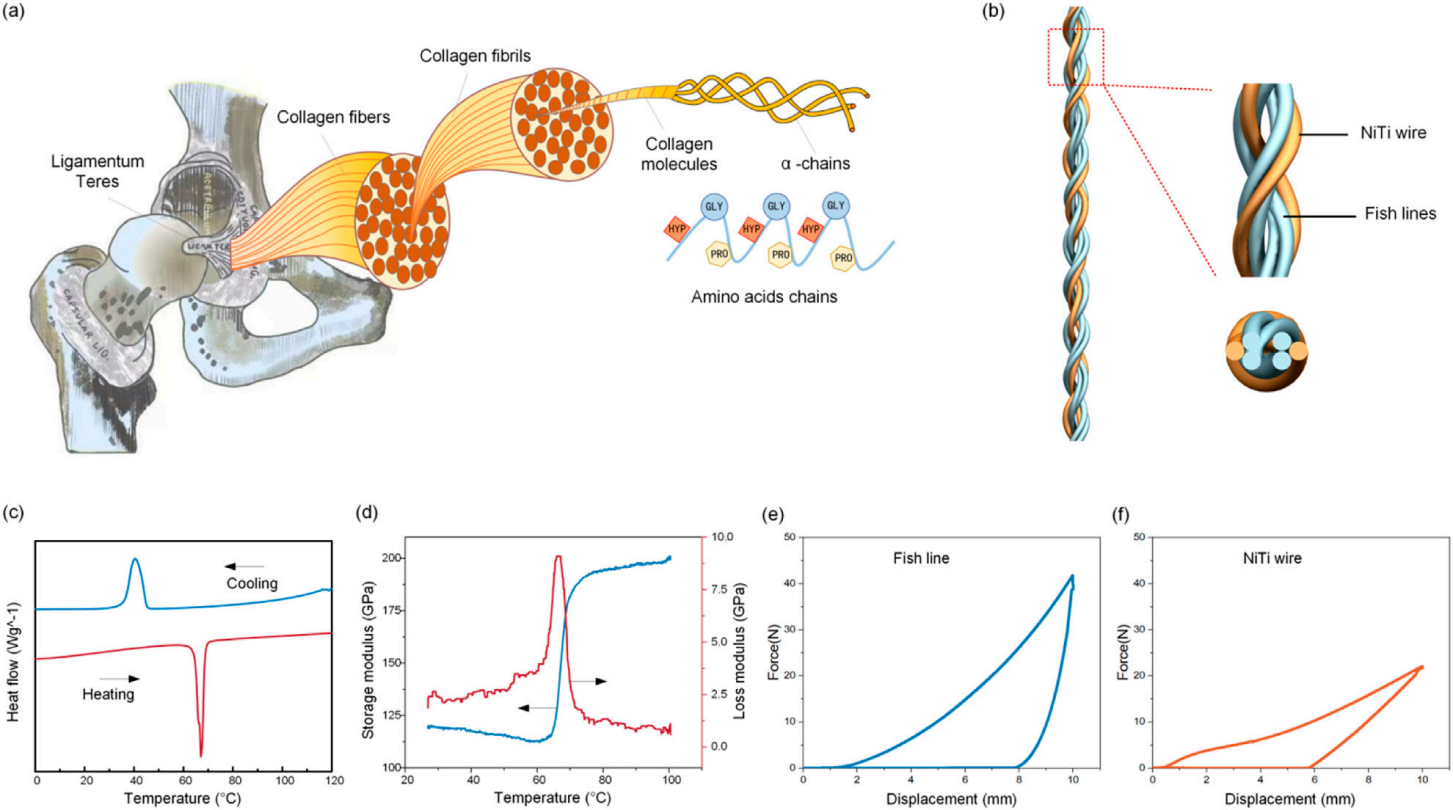
Self-healing ligament. **(a)** The hierarchical architecture of natural ligaments. **(b)** The hybrid artificial ligament integrating SMA wires and polyethylene fishing line. **(c)** The DSC measurements of the SMA material. **(d)** DMA characterization of the SMA material. Uniaxial tensile hysteresis testing of the **(e)** fish line and **(f)** SMA materials.

Natural ligaments possess a sophisticated hierarchical architecture composed of collagen fibrils, fibrous bundles, and macroscopic fascicles, which enables exceptional load-bearing capacity and self-recovery through dynamic collagen remodeling (Figure 1a). This study developed a 6-strand hybrid artificial ligament integrating Nickel-titanium shape memory alloy (SMA) wires and polyethylene (PE) fishing lines to mimic this structure (Figure 1b). The ligament was fabricated using a specialized disc braiding technique with a 1-over-1-under interlock configuration (6 carriers, 180° braid angle). After braiding, a customized fixture applied 5% strain at 60 °C for 12 h to stabilize the hierarchical structure via macromolecular chain relaxation and residual stress redistribution. These pre-tensioning parameters were adopted based on the authors’ previous work on 3D braided ligaments, which confirmed that this condition stabilizes the structure through macromolecular chain relaxation and residual stress redistribution without impairing the shape memory effect of SMA or the mechanical properties of PE fishing lines.

**FIGURE 2:**
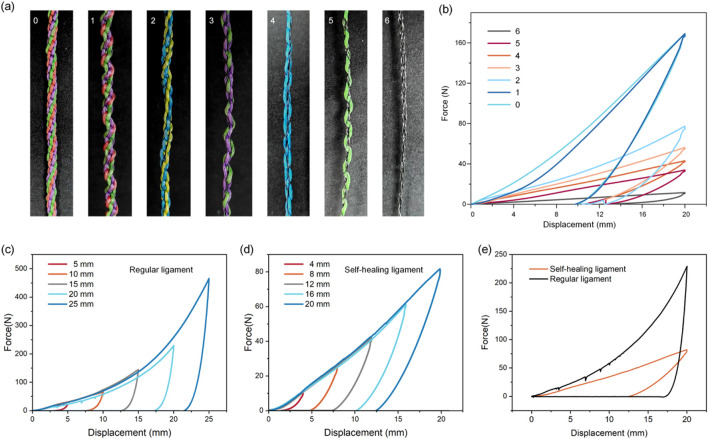
Mechanical properties of artificial ligaments with different numbers of SMAs. **(a)** Images of artificial ligaments with different numbers of SMA wires. **(b)** Hysteresis curves of artificial ligaments with different numbers of SMA wires. Hysteresis testing with different loading strains on **(c)** regular ligament and **(d)** self-healing ligament. **(e)** Comparison of energy absorption efficiency of regular and self-healing ligament.

Differential Scanning Calorimetry (DSC) measurements characterized the SMA wire’s thermal hysteresis (Figure 1c). The forward martensitic transformation had start (Ms) and finish (Mf) temperatures of 46.5 °C ± 0.8 °C and 27.2 °C ± 1.1 °C, while the reverse austenitic transformation occurred between 58.3 °C ± 0.6 °C (As) and 71.3 °C ± 0.9 °C (Af), forming a 24.8 °C thermal hysteresis window. Dynamic Mechanical Analysis (DMA) showed that during heating (30 °C–100 °C), the SMA’s storage modulus increased rapidly (forming an “S”-type curve) as martensite transformed to austenite, and Tan δ peaked during phase transformation (Figure 1d). Uniaxial tensile hysteresis tests at 25 °C revealed distinct mechanical behaviors: the PE fishing line reached 43 N at 5% strain (superior to the SMA wire’s 23 N at 5% strain), and the SMA exhibited characteristic flag-shaped hysteresis loops due to stress-induced martensitic transformation (Figures 2e,f).

### 3.2 Effect of the number of SMA wire on mechanical properties of artificial ligament

A parametric study replaced PE fishing line strands (0.50 mm diameter) with SMA wires (0.2 mm diameter) at 0%–100% substitution (1–6 strands), reducing the ligament diameter from 1.0 mm (pure PE) to 0.4 mm (pure SMA) and the maximum force by 15-fold (170 N–11 N) (Figures 2a,b).

Hysteresis tests on regular ligaments (no SMA) and self-healing ligaments (2 SMA wires) showed that hysteresis loop areas expanded with increasing strain (consistent with viscoelastic behavior) (Figures 2c,d). At the same strain, the self-healing ligament had a significantly lower energy dissipation ratio (Figure 2e), resulting from two factors: SMA’s inherently lower damping than viscoelastic polymers, and increased fiber packing density from SMA integration constraining fiber sliding friction (a key energy dissipation mechanism in textile-structured ligaments).

**FIGURE 3:**
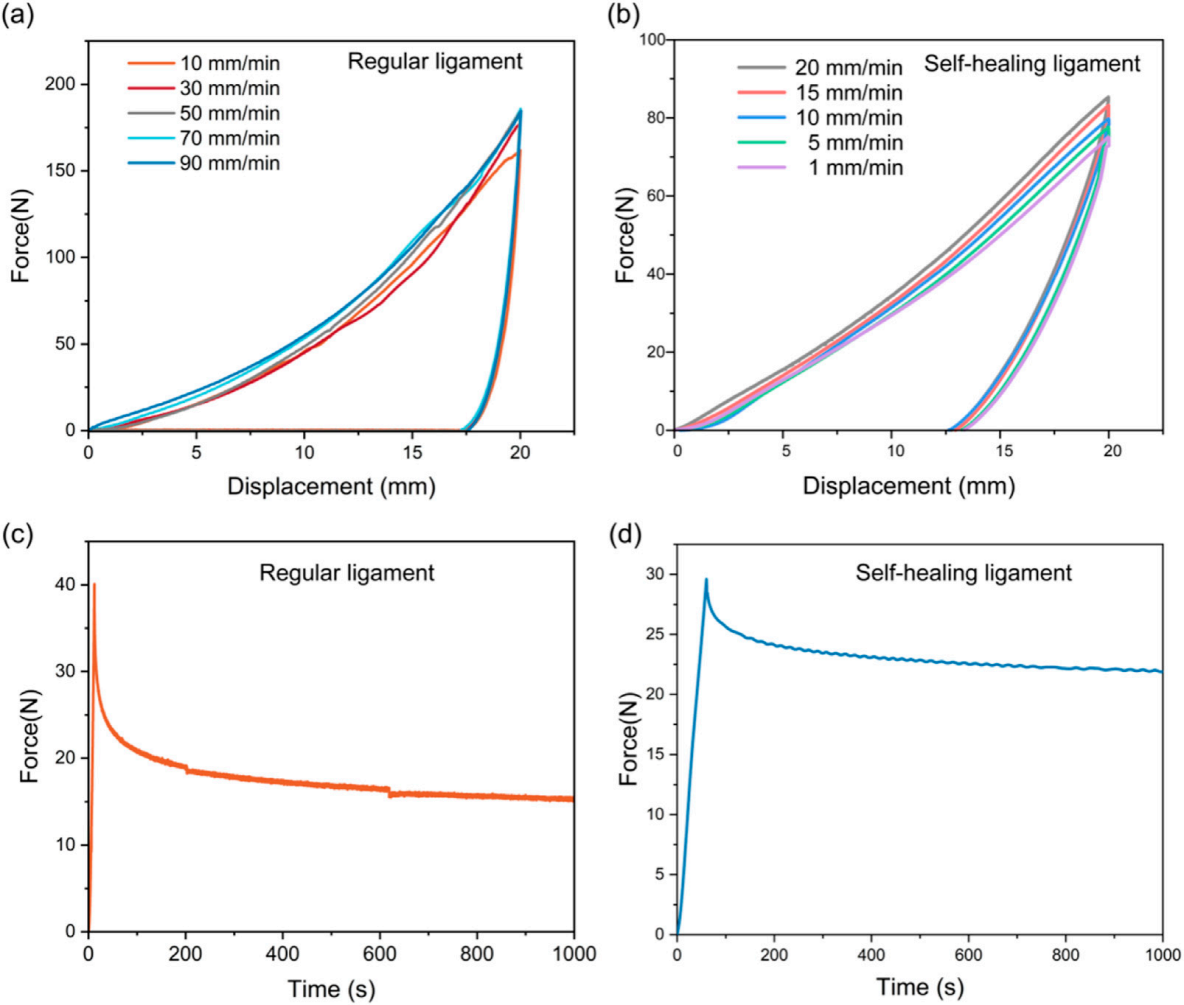
Viscoelastic characterization of regular and self-healing ligament. Hysteresis tests of **(a)** regular ligament and **(b)** self-healing ligament at varying strain rates. The stress relaxation behavior of **(c)** regular ligament and **(d)** self-healing ligament.

Cyclic loading tests at varying strain rates showed regular ligaments had larger hysteresis loops at higher rates (due to lagging polymer molecular chain relaxation) (Figure 3a). The self-healing ligament exhibited similar rate sensitivity but with a limited strain rate range (due to SMA’s high stiffness) (Figure 3b). Stress relaxation tests under 5% strain revealed the regular ligament’s progressive stress decay (polymer chain rearrangement), while the self-healing ligament retained ∼73% of initial stress after 1,000 s (vs. 37% for the regular ligament) (Figures 3c,d).

### 3.3 Electro-thermal recovery performance of pre-stretched self-healing ligaments

**FIGURE 4:**
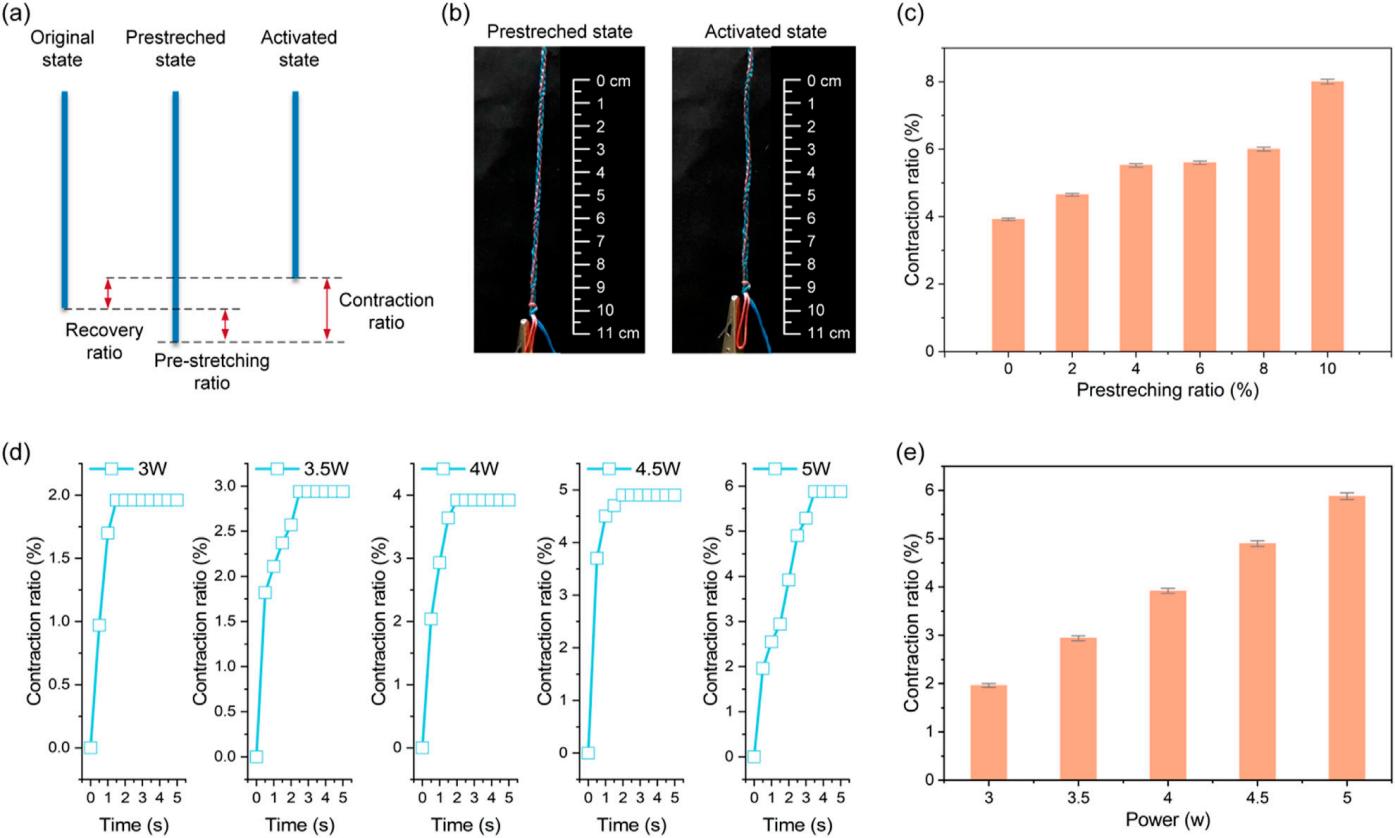
Electro-thermal recovery properties of the self-healing ligaments. **(a)** Diagram of the pre-stretching and contraction process. **(b)** The pre-stretched and activated state. **(c)** The maximum contraction rates under varying pre-stretching ratio. **(d)** The time-dependent contraction ratios under different power. **(e)** The maximum contraction rates under varying powers.

A two-stage protocol evaluated electro-responsive recovery: (1) pre-stretching specimens to defined elongations, clamping for 48 h, and confirming minimal shrinkage after unclamping; (2) applying controlled DC voltages and monitoring length until stabilization (Figures 4a,b).

At 5.5 W constant power, maximum contraction rates increased with pre-strain, but 6% pre-strain resulted in insufficient electrically activated contraction for full recovery (Figure 4c). Controlled electrical stimulation showed input power correlated with recovery: contraction ratios varied with power over time (Figure 4d), and increasing power from 3 W to 5.5 W (6 V DC) proportionally enhanced maximum contraction strain (Figure 4e). Below 3 W, SMA phase transformation was incomplete; at 4–5.5 W, linear temperature rise increased the austenite fraction and corresponding contraction strain.

### 3.4 Application of self-healing ligaments for modulating hip joint mobility

**FIGURE 5:**
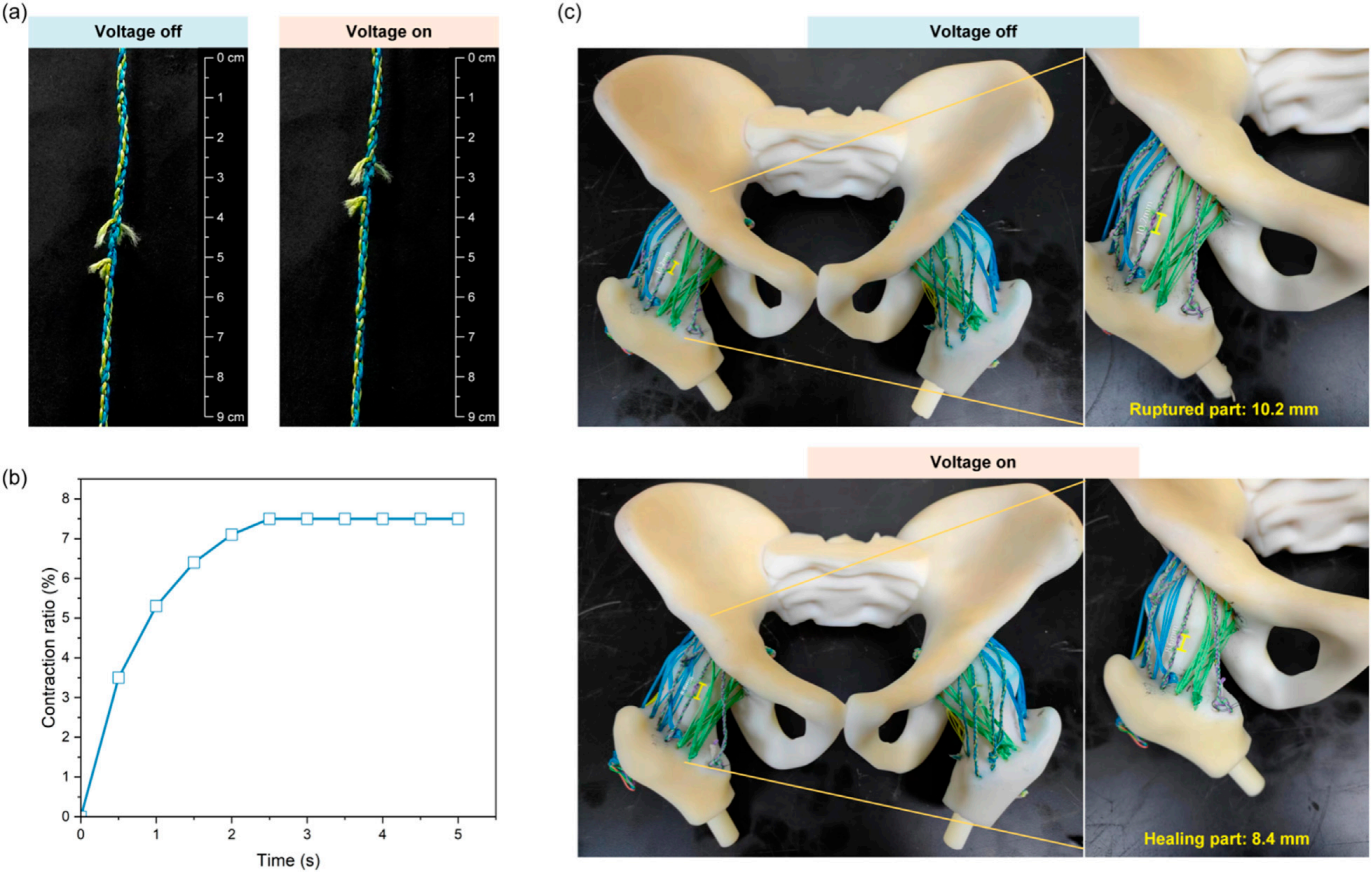
Demonstration of self-healing ligaments for recovering joint functionality. **(a)** Contraction behavior of the self-healing ligaments after rupture. **(b)** The time-dependent contraction ratios. **(c)** Hip joint model with self-healing ligaments.

After shearing off braided PE fishing lines (leaving only SMA), the self-healing ligament still contracted post-severe damage, with a stabilized contraction ratio of ∼7.5% of the original length (Figures 5a,b).

A 3D-printed artificial hip joint simulated iliofemoral, pubofemoral, and ischiofemoral ligaments (each with unbraided PE lines and one self-healing fiber). Eight infrared cameras analyzed 3D motion, showing the hip model’s range of motion (flexion/extension, internal/external rotation, adduction/abduction, translations) exceeded 95% of human hips in all directions, limiting excessive joint motion (Table 2).

**TABLE 2:** Comparison between the motion ranges of the artificial and human hip.

Motion	Axis	Human hip	Artificial hip		
			Initial	Ruptured	Healed
Rotations					
Adduction-Abduction	X	108°	106.4° ± 2.7°	134.2° ± 2.6°	101.6° ± 2.9°
Internal-External rotation	Y	108°	105.3° ± 2.3°	131.5° ± 2.5°	100.5° ± 2.8°
Extension-Flexion	Z	137°	135.6° ± 3.3°	169.2° ± 2.8°	126.2° ± 3.7°
Translations					
Posterior-anterior translation	X	11 mm	10.7 ± 0.3	12.0 ± 0.4	9.9 ± 0.6
Inferior-superior translation	Y	13 mm	12.5 ± 0.3	14.1 ± 0.5	11.8 ± 0.4
Medial-lateral translation	Z	9 mm	8.9 ± 0.2	10.1 ± 0.4	8.2 ± 0.6

Data are means ± s.d. For all the 10 trials (*n* = 10).

Another experiment cut self-healing ligament components (leaving SMA and intact unbraided PE) to test post-rupture laxity. SMA activation provided 1.8 mm hip contraction (17.6% of the 10.2 mm ruptured gap) (Figure 5c). Ruptured ligaments caused up to 26% excessive rotational motion and 12% excessive translational motion; post-healing, joint kinematics recovered to ∼95% of initial states (both rotations and translations), significantly improving laxity (Table 2).

## 4 Discussion

The development of a 3D-braided hybrid artificial ligament integrating SMA wires and PE fishing lines addresses longstanding hurdles in musculoskeletal engineering—specifically, the gap between synthetic materials that fail to replicate natural ligament mechanics, sustain long-term use, and enable self-repair. Conventional options often struggle with creep-induced loosening or poor biomechanical matching, while emerging self-healing designs frequently lack the robustness needed for practical application. This hybrid structure resolves these issues through intentional material synergy, with performance characteristics that align with the core needs of both clinical rehabilitation and broader engineering applications.

At its core, the ligament’s design draws from the structural intelligence of biological tissues. The high tensile strength of PE components—evident in their ability to withstand significant loads without failure—provides the foundational stability needed to support joint function, addressing the historical limitation of early synthetic ligaments that lacked sufficient load-bearing capacity (Lu et al., 2022; Sui et al., 2018). Complementing this, SMA wires introduce a dynamic dimension: their ability to undergo reversible phase transformation enables self-healing, moving beyond the static nature of traditional polymer-based alternatives. The 3D braided architecture further enhances this biomimicry, replicating the hierarchical fiber arrangement of natural ligaments to achieve the J-shaped stress-strain behavior critical for accommodating dynamic joint movements. Thermal characterization confirms the SMA’s stability at physiological temperatures, ensuring no unintended deformation during regular use—a key consideration for any material intended to interact with biological systems or sensitive mechanical structures (Lu et al., 2024).

The balance of mechanical stability and functional adaptability is a defining strength of the design. The configuration of SMA and PE components strikes a careful middle ground: it retains enough stiffness to prevent excessive joint motion, which is essential for post-injury recovery, while still enabling the controlled contraction needed for self-repair. This balance outperforms single-material alternatives, which often sacrifice either strength or adaptability. Additionally, the reduced energy dissipation ratio minimizes heat buildup during repeated use, extending the ligament’s lifespan and addressing a common failure point of materials subjected to cyclic loading (Zhi et al., 2022). Even under prolonged stress, the structure maintains a significant portion of its initial tension, ensuring consistent performance over time—a critical attribute for applications requiring reliability.

The electro-thermal recovery mechanism adds a layer of controllability that aligns with the vision of “smart” rehabilitation tools. The two-stage process of pre-stretching and electrical activation enables rapid, tailored repair: adjustments to pre-strain or power input allow customization for different injury severities, supporting personalized care approaches. The low power requirements—with a minimum threshold that avoids tissue damage while ensuring reliable activation—enhance practicality, making the design feasible for both implantable and external assistive devices. This efficiency stands in contrast to slower chemical self-healing systems, which often require complex triggers or lengthy recovery times.

Real-world validation of the ligament’s performance—through its integration into an artificial hip joint—confirms its ability to restore natural motion dynamics. The joint’s range of motion closely mirrors that of human hips, and its capacity to recover from simulated damage demonstrates the value of self-healing in maintaining long-term stability. This performance not only supports potential clinical use in ligament reconstruction but also extends to rehabilitation assistive devices, where flexibility and durability are equally important. Wearable joint supports, for instance, would benefit from the ligament’s ability to adapt to movement while retaining structural integrity (Park and Cho, 2017).

It is worth noting that recent advances in clinical-oriented ligament repair research, such as the piezoelectric nanomaterial (PENM)-based ACL repair strategy reported by Su et al., have highlighted the critical role of “material-ligament-central nervous system” synergy in improving repair outcomes. Their study demonstrated that PENM can generate microcurrents during joint motion to mimic proprioceptive signal afferents, thereby regulating the “cortex-basal ganglia-thalamus” neural circuit and promoting functional remodeling of brain regions related to motor control and sensory integration. While our 3D-braided hybrid ligament and PENM-based designs share the goal of addressing limitations in traditional ligament repair, they differ fundamentally in their core mechanisms and application priorities: PENM focuses on resolving proprioceptive deficits and central nervous system plasticity issues post-clinical injury, whereas our design prioritizes mechanical self-healing and structural stability—attributes that are equally critical for both clinical rehabilitation and broader engineering scenarios (Su et al., 2025).

From this foundation of medical and rehabilitation utility, the ligament’s potential in bionic tension-compression robots emerges as a natural extension of its core properties (Ren et al., 2021). The same graded stiffness that enables smooth joint motion in biological systems is critical for robotic joints requiring precise manipulation, addressing the challenge of coordinating rigid and flexible components in robotic design (Yan et al., 2022). The SMA-driven self-healing reduces the need for frequent maintenance—a significant advantage for robots operating in harsh or hard-to-access environments, such as pipeline inspection. Low energy consumption aligns with the power constraints of portable robotic systems, while the use of accessible, cost-effective materials addresses scalability concerns that often limit advanced robotic components (Attar et al., 2022).

The modularity of the design further supports this cross-disciplinary applicability. The structure that adapts to the complex motion of human hips can be reconfigured to suit robotic joints: its flexibility makes it suitable for continuum robots, while its load capacity benefits rigid-soft hybrid grippers. This transition is not a separate application but a reflection of the ligament’s inherent versatility—properties validated for medical use that also meet the demands of robotic engineering, where stability, adaptability, and durability are equally paramount.

Despite progress in mechanical matching and functional expansion of the bioinspired artificial ligament, the current design has three key limitations requiring future solutions: First, electrothermal self-healing depends on external DC power (6V DC). In clinical implantation, wired connections raise infection risks and restrict patient mobility (Santana et al., 2021); for robots, this limits untethered device range and battery life (Li et al., 2022). Miniature wireless power modules should be integrated to eliminate wires while ensuring stable 3–5.5 W output for SMA phase transformation. Second, long-term stability remains unvalidated. SMA may lose shape memory after repeated cycles (Hariri et al., 2022), PE may degrade/wear over time, and SMA-PE interfaces (relying on braided mechanical interlocking) may delaminate under prolonged loading. Accelerated aging tests are needed, along with plasma treatment to enhance interface adhesion, anti-degradation coatings for PE, and fatigue-resistant SMA alloys (Marzuki et al., 2020; Lee et al., 2021). Third, scaling production conflicts with customization. Dedicated braiding equipment allows some scalability, but adjusting parameters (strand count, angle) for patient/robotic needs increases costs. Modular braiding machines with replaceable molds and a “parameter-performance” database should be developed, and processes optimized to reduce unit costs—critical for robotic component cost-effectiveness.

In conclusion, this 3D-braided artificial ligament advances the goal of developing materials that bridge the gap between synthetic performance and biological function. Its ability to replicate natural mechanics, enable self-healing, and maintain durability validates its potential for clinical and rehabilitation use. Beyond this, its inherent properties—structural biomimicry, adaptive actuation, and cost-effectiveness—position it as a transformative component for bionic robots. This progression from addressing medical needs to unlocking engineering innovation underscores the value of drawing inspiration from biological systems to solve cross-disciplinary challenges.

## 5 Conclusion

This study proposes a biomimetic design strategy for self-healing artificial ligaments through the integration of SMA wires with fish line fibers. By modulating the number of embedded SMA wires, a substantial reduction—up to 15-fold—in maximum tensile force was achieved, ranging from 170 N to 11 N. Compared to the conventional ligament counterpart, the self-healing construct demonstrated a significantly lower energy dissipation ratio. It exhibited a rate-dependent response, while the range of strain rates tested was constrained by the inherent high stiffness of the SMA components. Additionally, the self-healing ligament with integrated SMA wires demonstrated markedly improved stress retention performance, maintaining approximately 73% of its initial stress after 1,000 s, as opposed to only 37% for the conventional design. Under constant power input, the maximum contraction rate of the self-healing ligament was positively correlated with the level of pre-stretch. Quantitative analysis revealed that at 6% pre-strain, the electrically induced contraction was insufficient to achieve full shape recovery. Furthermore, when input power was systematically increased from 3 W to 5.5 W, the maximum contraction strain exhibited a proportional increase. Importantly, artificial hip model tests demonstrated that these ligaments could offer improved joint laxity after severe damage, restoring joint kinematics to ∼95% of the pre-injury state in both rotational and translational motions. In conclusion, the proposed self-healing ligament maintains mechanical integrity across multiple deformation cycles and provides functional recovery, suitable for clinical applications and rehabilitation devices in joint injury treatments.

## Data Availability

The raw data supporting the conclusions of this article will be made available by the authors, without undue reservation.
